# Editorial: The state of the art in head and neck cancer and carcinogenesis translational research

**DOI:** 10.3389/froh.2023.1266272

**Published:** 2023-08-08

**Authors:** Felipe Nör, Fernanda Visioli

**Affiliations:** ^1^ Department of Periodontics and Oral Medicine, University of Michigan School of Dentistry, Ann Arbor, MI, United States; ^2^ Oral Medicine Department, School of Dentistry, Universidade Federal do Rio Grande do Sul (UFRGS), Porto Alegre, Brazil; ^3^ Experimental Center Research, Hospital de Clínicas, Porto Alegre, Brazil

**Keywords:** head and neck cancer, carcinogenesis, cancer therapy, translational research, cancer prevention & control

**Editorial on the Research Topic** The state of the art in head and neck cancer and carcinogenesis translational research

Head and neck cancer (HNC) is the seventh most common malignancy with an annual global incidence of 1.1 million new cases (1). Research has been instrumental in improving our understanding of the biology and behavior of this particular subset of cancers. However, the translation of data to the patient has not always been a reality, as evidenced by the marginal improvement in clinical outcomes over time. Translational research aims to narrow this gap between research and patient care. This Research Topic highlights mini reviews, reviews, and original research that approach the issue of HNC and carcinogenesis from a translational perspective.

To improve the mortality rates associated with HNC, it is imperative to enhance awareness and early detection efforts. Zabala et al. demonstrated that an early detection program targeting high-risk individuals could enhance the cost-effectiveness of programs while optimizing resource allocation. The program involved 324 asymptomatic patients aged over 50 years presenting with either one major risk factor (tobacco or alcohol consumption) or two minor risk factors, which include family history of HNC, occupational exposure, poor oral hygiene, history of Human Papillomavirus, or chronic inflammatory processes of the aerodigestive tract. A clinical screening was conducted, resulting in a detection rate of 1.2% for neoplasia and 4.6% for pre-neoplastic lesions within the targeted population. From a translational perspective, this research highlights the potential of screening programs as a means for the early diagnosis of potentially malignant disorders and HNC.

Early detection of potentially malignant disorders, such as leukoplakia, holds the potential for implementing preventive measures, including smoking and alcohol cessation programs. Furthermore, as emphasized in the review conducted by Palma et al. secondary prevention emerges as a promising strategy to impede the progression of these disorders into cancer. The review identified the main systemic and topical agents for chemoprevention and examined the results of relevant trials. By comprehensively synthesizing the available evidence, the review provided an in-depth overview of the current landscape of chemoprevention for oral leukoplakia. From a translational perspective, though there is currently no specific agent considered as the standard of care for oral cancer chemoprevention, the authors have identified promising treatments that require further validation. Furthermore, several strategies were proposed to improve the search for an ideal chemopreventive protocol.

Cancer prevention may also involve the implementation of protective agents. Fan explored epidemiological evidence suggesting that the consumption of caffeinated coffee could potentially reduce the risk of developing HNC. However, the exact mechanisms behind this protective effect remain uncertain. To bridge this knowledge gap, Fan proposed a hypothesis that offers a potential explanation for this observed phenomenon. Both *in vitro* and *in vivo* studies have demonstrated that caffeine has the capability to inhibit the activation of the NLRP3 inflammasome, a protein complex responsible for initiating inflammatory signaling pathways in response to various stimuli. Previous research has clearly indicated the significant roles of NLRP3 inflammasome activation in promoting carcinogenesis and tumor growth in oral cancer. From a translational perspective, if confirmed, this hypothesis could have valuable implications potentially guiding the development of new approaches for cancer prevention.

Transitioning from preventive measures to patient screening methods, Ghita et al. analyzed the expression profile of an inflammatory cytokine panel in the plasma of patients and its correlation with two distinct histological patterns of HNC (i.e., inflamed and immune-excluded). Previous data from the same group showed that high levels of the soluble immune biomarker Semaphorin 4D (Sema4D) were correlated with a higher expression of osteopontin (OPN) in tumor tissues, characterizing a positive feedback loop. In the present study, higher Sema4D and OPN expression levels were observed in the immune-excluded histological pattern of HNC. Notably, elevated levels of OPN have been associated with resistance to therapy and poor patient survival. From a translational perspective, the identification of cytokine levels in plasma samples might represent an adjuvant tool for the stratification of HNC patients that could benefit from therapeutic cytokine inhibition.

The hierarchical model of carcinogenesis centered on the cancer stem cell hypothesis has certainly transformed our overall understanding of cancer biology, creating novel approaches to treat this disease. Among the various intra- and extracellular biomarkers utilized for the identification of cancer stem cells (CSC), the review by Herzog et al. focused on Bmi-1 due to its significant participation in cancer stem cell regulation. Bmi-1 is involved in key signaling pathways that ultimately drive tumorigenesis and cancer progression. Notably, Bmi-1^+^ CSC were found to be resistant to the most common therapeutic modalities for HNC (i.e., chemotherapy and radiotherapy), favoring tumor progression and metastases and, consequently, poor patient outcomes. From a translational perspective, the encouraging data from *in vitro* studies targeting Bmi-1 in HNC combined with the positive results in other tumor types support future preclinical and clinical studies to determine the role of this therapeutic strategy in HNC patients.

One of the current dilemmas in oral medicine and oral pathology is the inability to predict when or whether a certain lesion will undergo malignant transformation. Emerging evidence suggests that CSC may also play a contributory role in the progression of oral premalignant lesions (e.g., leukoplakia). The review by Polverini et al. explored the proposed mechanism by which CSC drive the progression of premalignant lesions to more advanced stages of dysplasia and, ultimately, to cancer. Notably, data suggest that CSC do not act alone in this process. In fact, they depend on the exchange of mediators secreted by adjacent endothelial and inflammatory cells (i.e., macrophages). From a translational perspective, the development of therapeutic strategies targeting CSC as well as their niche of support may not only impact HNC treatment but, perhaps more importantly, its prevention.

This Research Topic compiles a series of comprehensive reviews and original research studies focusing on crucial aspects in HNC: from prevention and early detection, to patient screening and treatment. Taken together, this research effort represents an important step towards the goal of translating knowledge for the benefit of patients.
